# Influence of b2 adrenergic receptor polymorphism (rs1042713 and rs1042714) on anthropometric, hormonal and lipid profiles in polycystic ovarian syndrome

**DOI:** 10.5937/jomb0-26183

**Published:** 2021-01-26

**Authors:** Maha H. Daghestani, Maha Omair, Mazin Daghestani, Sonya S. Abdel-Razeq, Namik Kaya, Arjumand Warsy

**Affiliations:** 1 King Saud University, Center for Female Scientific and Medical Colleges, Department of Zoology, Saudi Arabia; 2 King Saud University, College of Science, Department of Statistics and Operations Research, Saudi Arabia; 3 Umm-Al-Qura University, Department of Obstetrics & Gynecology, Saudi Arabia; 4 Yale University School of Medicine, Department of Obstetrics, Gynecology, and Reproductive Sciences, Division of Maternal-Fetal Medicine, New Haven, CT, USA; 5 King Faisal Specialist Hospital and Research Center, Department of Genetics, Riyadh, Saudi Arabia; 6 King Saud University, Center for Female Scientific and Medical Colleges, Central Laboratory, Riyadh, Saudi Arabia

**Keywords:** beta-adrenergic receptor, leptin, lipids, polycystic ovary syndrome, single nucleotide polymorphism, pojedinačni nukleotidni polimorfizam, policistični sindrom ovarija, lipidi, leptin, beta-adrenergični receptor

## Abstract

**Background:**

Polycystic ovarian syndrome (PCOS) is a frequently encountered disorder. This study aimed to identify polymorphisms in ADRB2 in Saudi PCOS development and to study its influence on lipids, hormones, and anthropometric parameters.

**Methods:**

Saudi females (100) suffering from PCOS and healthy controls (100) were investigated. The estimation of cholesterol, triglycerides, high-density lipoprotein (HDL-C), low-density lipoprotein (LDL-C), plasma glucose, leptin Insulin, and ghrelin were carried out. The DNA was extracted, and* ADRB2* fragment carrying the exon 1 was amplified and sequenced.

**Results:**

The waist, W/H ratio, lipids, glucose, and insulin were significantly higher in the obese PCOS compared to the normal weight group. The leptin and ghrelin were not different. Two single nucleotide polymorphisms (SNPs): rs1042713 (Arg16Gly; A>G) and rs1042714 (Gln27Glu; C>G) were identified. The genotype and allele frequency of rs1042713 did not differ in the total PCOS and normal weight, and obese PCOS compare to the controls. However, rs1042714 was significantly associated with PCOS development, where the minor G allele was protective against PCOS development.

**Conclusions:**

The rs1042714 polymorphism of the *ADRB* associates with PCOS development in Saudis, while rs1042713 does not. However, the GG genotype of rs1042713 associates significantly with elevated BMI, waist, hip, W/H, and leptin, and decreased ghrelin. On the other hand, rs1042714 genotypes do not associate with any abnormality except the homozygous GG have higher triglycerides and lower HDL-C. Interestingly, glucose showed different correlation patterns in individuals carrying different genotypes of the two studied SNP, indicating clearly that the metabolic responses to a normal nutrient are significantly influenced by the genotypes of the SNPs in *ADRB2*.

## Abstract (sr)

**Uvod:**

Sindrom policističnih ovarija (PCOS) je učestao problem. Ovo izučavanje ima za cilj da identifikuje polimorfizam u ADRB2 i PCOS u razvoju kod Saudijskih žena i njegov uticaj na lipide, hormone i antropometrijske parametre.

**Metode:**

Proučavano je 100 Saudijskih žena sa PCOS i 100 zdravih žena kao kontrola. Određivani su holesterol, trigliceridi, lipoproteini velike gustine (HDL-C), lipoproteini male gustine (LDL-C), glukoza, leptin, insulin i grelin. Izolovana je DNK i *ADRB2* fragment koji nosi ekson 1 koji je amplifikovan i sekvencioniran.

**Rezultati:**

Struk, W/H odnos, lipidi, glukoza i insulin su bili značajno viši u gojaznih PCOS u poređenju sa grupom koja je imala normalnu težinu. Leptin i grelin se nisu razlikovali. Identifikovana su dva pojedinačna nukleotidna polimorfizma (SNPs) rs1042713 (Arg16 Gly; A>G) i rs1042714 (Gln27Glu; C>G). Genotip i alelna frekvencija rs1042713 nije se razlikovala kod svih PCOS sa normalnom težinom i gojaznih PCOS u poređenju sa kontrolama. Međutim, rs1042714 je značajno bio udružen sa razvojem PCOS, pri čemu je manji G alel bio zaštitnik za razvoj PCOS.

**Zaključak:**

Polimorfizam rs1042714 ADRB je udružen sa razvojem PCOS kod Saudijskih žena za razliku od polimorfizma rs1042713. Međutim, genotip rs1042713 je značajno praćen sa povećanjem BMI, struka, butina, W/F i leptina dok je grelin smanjen. S druge strane, rs1042714 genotip nije udružen sa bilo kojim poremećajem izuzev što kod homozigota GG postoji povećanje triglicerida i sniženje HDL-C. Zanimljivo je da glukoza pokazuje različitu korelaciju u osoba koje nose različite genotipe u dva proučavana SNP, što ukazuje na metabolički odgovor na normalne nutrijente i značajan uticaj genotipova SNPs u ADRB2.

## Introduction

Polycystic ovary syndrome (PCOS) is a common multiple endocrine dysfunction disorder, which is characterized by heterogeneity in its clinical presentation, heritability, the age of onset and severity of associated complications [1]. Ovaries are polycystic, and other frequent manifestations include oligomenorrhea or amenorrhea, hirsutism, hyperandrogenism, and infertility [2]. Majority of the women suffering from PCOS have insulin resistance and its associated comorbidities such as metabolic syndrome, dyslipidaemia, glucose intolerance, and diabetes. Several factors show association with PCOS development and include types 1 and 2 diabetes mellitus, gestational diabetes, obesity and genetic factors. Obesity is considered as a frequently occurring factor associated with an increased risk of PCOS, even in children [3]. In several women, a history of weight gain often precedes the development of the clinical features of PCOS. A PCOS prevalence of 28.3% was reported in a group of women referred for assistance with weight loss. It was shown in another study that changes made in lifestyle that lead to amelioration of body weight, abdominal fat, and insulin resistance improves the clinical manifestations of PCOS [4].

Due to the close association between obesity and PCOS, we hypothesized that genes which have been implicated in the development of obesity may play a role in PCOS and may be involved in the development of the associated comorbidities. Several studies have shown an association between polymorphisms in the beta-adrenergic receptor (*ADRB2*) gene and obesity and obesity-related disorders, including metabolic syndrome, hyperlipidaemias, cardiovascular disease, hypertension, etc., while others have shown contradictory results. Beta-adrenergic receptor (ADRB) occurs as three subtypes which are distributed in different tissues, and play a key role in the mobilization of lipids through lipolysis in human adipose tissue *in vivo* [5]. A study on Japanese women showed that rs1042714 (C>G) in *ADRB2* is linked to PCOS [6]. This is a non-synonymous mutation, which is the substitution of the 27^th^ amino acid i.e. glutamine by a glutamic acid. Another polymorphism that has been investigated in several studies is rs1042713 (A>G), which is also a non-synonymous change resulting in the substitution of arginine at amino acid 16 by glycine. In a study of four polymorphisms in *ADRB2* gene, the haplotype CCGG constructed from these were shown to have a protective effect against the development of insulin resistance and metabolic syndrome in PCOS [7]. We designed this study to investigate the association between two common polymorphisms (rs1042713 and rs1042714), in the *ADRB2* gene and anthropometric, hormonal and lipid abnormalities in Saudi women suffering from PCOS. Since no such studies have been carried out on PCOS patients, we expected to identify genetic factors that may influence the management of PCOS. In addition, we studied whether the genotypes influence correlation between different parameters and we selected glucose level to study this influence. Glucose abnormalities occur frequently in PCOS and may lead to different complications. In this paper, we present our findings of the association between genotypes of the two SNPs and the studied parameters in the Saudi women suffering from PCOS.

## Materials and Methods

### Samples

Ethical approval was obtained from the Institutional Review Board (IRB), Umm Al-Qura University, Makkah, Saudi Arabia (IRB No. 235). The samples were collected for a period of one year from July 2017. The study group included women volunteers suffering from PCOS, and healthy controls attending the out-patient clinic at the Department of Obstetrics and Gynecology, and Private Medical Centers, Makkah, Kingdom of Saudi Arabia. The diagnosis of PCOS was based on the criteria set by the Rotterdam consensus [8]. The health status of the control group was determined by examination of their medical history and by a physical and pelvic exam. The controls had regular ovulatory cycles (as confirmed by transvaginal ultrasound and plasma progesterone assay during the luteal phase of the cycle), normal ovarian morphology on ultrasound, and normal levels of androgens. Exclusion criteria were as follows: pregnancy, hyperprolactinemia, hypothyroidism, congenital adrenal hyperplasia, Cushing's syndrome, current or previous (within the last six months) use of oral contraceptives, anti-androgens, ovulation induction agents, anti-diabetics, glucocorticoids, anti-obesity drugs or other hormonal drugs. None of the patients and controls was affected by metabolic, cardiovascular, neoplastic disorder and or other concurrent medical illnesses such as diabetes, renal disease, and hepatic disorders. All the subjects had normal physical activity, and none of them were smokers. The study protocol was explained to the patients and the controls, and each participant was required to sign an informed consent form before inclusion in the study.

### Anthropometric measurements

On a prearranged day, each PCOS patients and control attended the clinic following overnight fasting. Age and family history were recorded. The PCOS and control groups were matched for age (±2 years). Weight and height were measured by routine methods, and Body Mass Index (W (Kg)/H^2^ (m^2^)) was calculated. The circumference of the waist (the narrowest measurement between the lower costal margins and the iliac crest) and hip (the maximum circumference at the level of the femoral trochanters) were measured when the women were in the standing position for obtaining the waist-hip ratio (WHR).

### Extraction of blood samples

A venous peripheral blood sample was drawn by venipuncture between 8.00-9.00 a.m., during the early follicular phase (2nd or 3rd day), following an overnight (12 hrs.) fast. Two ml blood was collected in EDTA tubes for DNA extraction, two ml in aprotinin tubes (500 kU/mL; Trasylol; Bayer Corp., Lever kusen, Germany) for total ghrelin estimation, one ml in fluoride tubes (gray top) for glucose estimation and two ml in plain tubes for the biochemical analysis.

All samples were immediately centrifuged, and the serum or plasma was stored at -80 °C until further analysis. The genomic DNA was extracted from whole blood leucocytes using Gentra Systems Kit (Minneapolis, MN, cat # D5500).

### Estimation of biochemical and hormonal parameters

The serum lipids (cholesterol, triglycerides, high-density lipoprotein-cholesterol (HDL-C), and low-density lipoprotein cholesterol (LDL-C)) were estimated by enzymatic methods using commercial kits (Boehringer Mannheim). The plasma glucose level was determined by the glucose oxidase method on a Beckman Glucose Analyzer (Fullerton, CA). The leptin and ghrelin levels were estimated using Leptin ELISA Kit (Phoenix Pharmaceuticals Inc., Belmont, CA, USA), and ghrelin (human) enzyme immunoassay kit (EIA) (Phoenix Pharmaceuticals Inc., Belmont, CA, USA). Insulin level was determined by the electrochemiluminescence immunoassay »ECLlA« on a Roche Elecsys 1010/2010 and MODULAR ANALYT-ICS E170 (Elecsys module) immunoassay Analyzers (Roche Diagnostic, Mannheim, Germany).

### Genotyping of ADRB2 gene polymorphism

The DNA fragment carrying the codons 16 and 27 of *ARDB2* gene was amplified by polymerase chain reaction (PCR) using the following forward (F) and reverse (R) primers (F: 5'-AAGCTGAGTGT-GCAGGACGA-3 and R: 5'-AGACGCTCGAACTTG-GCAAT-3 ). The conditions used for the PCR amplification consisted of an initial denaturation step at 95 °C for 15 minutes, and 34 cycles of 95 °C for 1 minute (denaturation), 64 °C for 1 minute (annealing) and 72 °C for 1 minute (extension), with a final extension of 10 minutes at 72 °C. The PCR product was subjected to agarose gel electrophoresis, and a 353 bp fragment was obtained. It was subjected to nucleotide sequencing using the ABI Big Dye Terminator protocol on ABI 3100 Avant Genetic Analyzer.

### Statistical analysis

All statistical analyses were conducted using the statistical software SPSS (Version18). The data for the total population were analyzed, and based on the BMI, two groups were formed: normal weight group (BMI <24.9) and obese group (BMI >30). The data were separately analyzed, and the results are presented as mean ± SEM. The results obtained for the control group and the PCOS group was compared using the independent Student's-test. A frequency distribution analysis was performed. The genotypes and alleles were assessed, and frequencies were calculated. The significance of the difference in the results of different groups was obtained using the chi-square test. Frequencies of the different genotypes and alleles in different groups and between control and PCOS were compared. Genotype and allelic frequencies in patients and controls were computed for Hardy-Weinberg equilibrium, which was investigated using the website http: //ihg2.helmholtz-muenchen.de/cgi-bin/hw/hwa1.pl. Odds ratio and their 95% confidence intervals (CIs) were obtained using http: //ihg2.helmholtz-muenchen.de/cgi-bin/hw/hwa1.pl. A probability value (p) ≤ 0.05 was considered statistically significant. The entire study groups were divided into three subgroups each, based on the genotypes of rs1042713 and rs1042714, and the phenotypic characteristics and levels of biochemical and hormonal parameters were calculated in the groups. A comparative analysis was performed using the ANOVA test. Correlation analyses were carried out, and Pearson's correlation coeficient (r) and the P-value were obtained (for all study groups the power of the study ranged between 0.65-0.90).

## Results

There were 39 normal weight and 61 obese PCOS females, and an equal number of age-matched controls were recruited. The results obtained in the normal weight (BMI: ≤24.9 kg/m^2^) and obese (BMI: ≥30.0 kg/m^2^) individuals in the controls and PCOS population were separately analyzed and compared. The anthropometric data of normal weight, obese, and total PCOS and controls are presented in Table 1. Age and BMI in total PCOS matched with that in the control group. The waist was significantly higher in the normal weight PCOS compared to the normal weight controls, but the obese PCOS group and obese controls did not show a significant difference. Hip circumference was lower in the PCOS group compared to the control group, and the difference was significant in the obese and total groups, but not in the normal-weight group.

**Table 1:** Anthropometric characteristic of normal weight and obese Controls and PCOS females Abbreviations – BMI: body mass index; W/H: waist/hip ratio. *The difference in the results of normal weight and obese subjects in the controls and PCOS is statistically significant.

Variable		Control (n=100) Mean±SEM	PCOS (n=100) Mean±SEM	P-value (PCOS compared to control)
Age (years)	Normal weight	25.31±0.801	23.79±0.721*	0.164
	Obese	24.26±0.720	25.84±0.418*	0.062
	Total	24.67±0.539	25.04±0.0.39	0.579
BMI (kg/m^2^)	Normal weight	22.172±0.234*	22.813±0.252*	0.066
	Obese	34.53±0.738*	33.136±0.53*	0.128
	Total	29.71±0.759	29.11±0.608	0.538
Waist	Normal weight	69.539±0.842*	76.821±1.494*	0.0001
	Obese	95.62±1.918*	94.13±1.549*	0.546
	Total	85.45±1.761	87.38±1.393	0.391
Hip	Normal weight	98.00±0.984*	96.487±1.095*	0.307
	Obese	117.95±1.823*	108.93±1.395*	0.0001
	Total	110.17±1.526	104.08±1.128	0.002
WH ratio	Normal weight	0.708±0.007	0.794±0.0116	0.0001
	Obese	0.8098±0.007	0.8649±0.0091	0.0001
	Total	0.770±0.0071	0.837±0.00795	0.0001

Interestingly, the WHR ratio was significantly higher in normal weight, obese, and total PCOS group, compared to the control groups, and in each case, the results were significantly different. Results in obese PCOS were also compared with the results in normal-weight PCOS, and results in normal-weight controls were compared to obese controls. Differences were significant in all parameters except age and WHR, in both groups.

Table 2 presents the results of the lipid profile, leptin, ghrelin, insulin, and glucose in the normal weight and obese PCOS and control groups. These parameters were significantly elevated in the total PCOS group compared to the control group, except for HDL-cholesterol, which was reduced considerably and leptin and ghrelin, which were not different. When the normal-weight PCOS and controls were separated, the difference persisted in the normal weight group. However, between the obese PCOS and obese controls, the significance was lost for triglyceride, HDL-cholesterol, and fasting insulin, while other parameters continued to be higher in the PCOS. Within the PCOS, the obese PCOS patients had significant elevation of cholesterol, triglycerides, LDL-cholesterol, and leptin and a decrease in ghrelin level compared to the lean PCOS. While in the control group, all parameters were elevated in the obese controls, except for HDL-cholesterol and ghrelin, which were significantly decreased.

**Table 2:** Comparisons of lipids and hormonal parameters amongst normal weight and obese Control and PCOS females *The difference in the results of normal weight and obese is statistically significant.

Variables		Control (n=100) Mean±SEM	PCOS (n=100) Mean±SEM	P-value
Cholesterol (mmol/L)	Normal weight	3.456±0.066*	3.931±0.068*	0.0001
	Obese	3.866±0.073*	4.684±0.114*	0.0001
	Total	3.706±0.055	4.390±0.083	0.0001
Triglyceride (mmol/L)	Normal weight	0.713±0.037*	0.902±0.055*	0.006
	Obese	1.050±0.058*	1.169±0.052*	0.128
	Total	0.919±0.041	1.065±0.041	0.012
HDL cholesterol (mmol/L)	Normal weight	1.411±0.048*	1.063±0.044	0.0001
	Obese	1.11±0.039*	1.15±0.044	0.560
	Total	1.229±0.033	1.114±0.032	0.014
LDL cholesterol (mmol/L)	Normal weight	1.267±0.055*	2.228±0.045*	0.0001
	Obese	2.120±0.079*	2.589±0.102*	0.0001
	Total	1.787±0.067	2.448±0.067	0.0001
Leptin (ng/mL)	Normal weight	13.308±0.592*	13.744±0.495*	0.574
	Obese	40.27±2.613*	34.43±1.755*	0.066
	Total	29.76±2.079	26.36±1.484	0.185
Fast ghrelin (ng/mL)	Normal weight	0.524±0.013*	0.524±0.018*	0.982
	Obese	0.323±0.014*	0.338±0.014*	0.446
	Total	0.401±0.14	0.411±0.14	0.641
Fasting insulin (pmol/L)	Normal weight	53.482±2.54*	83.741±10.29	0.007
	Obese	94.52±5.36*	106.94±6.72	0.151
	Total	78.495±3.958	97.895±5.820	0.006
Fasting glucose (mmol/L)	Normal weight	4.559±0.061*	4.972±0.062	0.0001
	Obese	4.913±0.064*	5.107±0.058	0.028
	Total	4.775±0.0488	5.054±0.043	0.0001

Sequence analysis showed the presence of only two polymorphic single nucleotide variations: rs1042713 (A>G) and rs1042714 (C>G). The geno type and allele frequencies of both SNPs were calculated in the PCOS patients and controls, and the results are presented in Table 3. For both SNPs, the Hardy-Weinberg equilibrium was tested for the study group. No difference was seen in the genotype and allele frequencies of rs1042713 in the PCOS and control group. However, rs1042714 showed an almost significant difference in allele frequencies of the C>G transversion. The mutant G allele was protective against PCOS development (OR=0.662; p=0.054), and the frequency of wild type C allele and genotype CC were higher in PCOS. The normal weight and obese PCOS and controls were separated, and the genotype and allele frequencies of both SNPs, were investigated. The results of genotype and allele frequencies, χ^2^, and p values are presented in Table 4. The rs1042713 genotypes and alleles show no significant difference between the obese or normal weight groups. However, for rs1042714, the C allele and genotype CC was higher in the PCOS compared to the controls in the normal weight group, but not in the obese group.

**Table 3:** Genotype and allele frequencies of the rs1042713 and rs1042714 in ARDB1 in the total PCOS females compared to the healthy controls

Genotype/Allele	Control Group (100)	PCOS group (100)	OR (95%CI)	χ^2^	p-value
	N (Frequency %)	N (Frequency %)			
Genotype frequency (rs1042713 (Arg16Gly))					
AA	55(55.0)	54(54.0)	Reference		
AG	23 (23.0)	23(23.0)	1.019 (0.511–2.029)	0.00	0.958
GG	22(22.0)	23(23.0)	0.939 (0.486–1.834)	0.03	0.859
** Allele frequency **					
A	133(66.5)	131(65.5)	Reference		0.833
G	67(33.5)	69(34.5)	0.956 (0.632–1.447)	0.045	
** Genotype frequency (rs1042714 (Gln27Glu)) **					
CC	56 (56)	65 (65)	Reference		
CG	14 (14)	14 (14)	0.862 (0.379–1.961)	0.13	0.722
GG	30 (30)	21 (21)	0.603 (0.311–1.170)	2.26	0.132
** Allele frequency **					
C	126 (63)	144 (72)	Reference		0.054
G	74 (37)	56 (28)	0.662 (0.434–1.009)	3.692	

**Table 4:** Genotype and allele frequencies of the rs1042713 (Arg16Gly) polymorphism in *ADRB2* gene, in normal weight and obese controls and PCOS females

Group		No.	Genotype No. (%)				Allele Frequency No. (%)		
Frequency of rs1042713 (Arg16Gly) in normal weight and Obese									
Group		No.	AA	AG	GG	χ^2 ^(p)	A	G	χ^2^ (p)
Normal weight	Control	39	28 (71.8)	7 (17.9)	4 (10.3)	1.49 (0.475)	63 (80.8)	15 (19.2)	1.3 (0.254)
	PCOS	39	23 (59.0)	11 (29.2)	5 (12.8)		57 (73.1)	21 (26.9)	
Obese	Control	61	27 (44.3)	16 (26.2)	18 (29.5)	0.85 (0.655)	70 (57.4)	52 (42.6)	0.271 (0.603)
	PCOS	61	31 (50.8)	12 (19.7)	18 (29.5)		74 (60.7)	48 (39.3)	
** Frequency of rs1042714 (Gln27Glu) in normal weight and Obese **									
** Group **		** No. **	** CC **	** CG **	** GG **	** χ^2^ (p) **	** C **	** G **	** χ^2^ (p) **
Normal weight	Control	39	21 (53.8)	5 (12.8)	13 (33.3)	3.44 (0.179)	47 (60.3)	31 (39.7)	4.238 (0.0395)
	PCOS	39	26 (66.7)	7 (17.9)	6 (15.4)		59 (75.6)	19 (24.4)	
Obese	Control	61	35 (57.4)	9 (14.8)	17 (27.9)	0.591 (0.744)	79 (64.8)	43 (34.2)	0.67 (0.413)
	PCOS	61	39 (63.9)	7 (11.5)	15 (24.6)		85 (69.7)	37 (30.3)	

In an attempt to investigate the influence of the different genotypes of the two studied polymorphisms on the anthropometric measures, hormonal and lipid profiles, the total study population was separated into three groups based on the genotypes they carried and the hormonal and lipid profiles were calculated in each genotype. The results of the different parameters in the three genotypes of rs1042713 (AA, AG, and GG) are presented in Table 5, and Table 6 presents the results in the three genotypes of rs1042714 (CC, CG, and GG). Table 5 shows that the subjects carrying GG genotype of rs1042713, i.e., Gly16 in the homozygous state, had a higher BMI, waist, and hip circumference, W/H ratio, fasting glucose and plasma leptin. At the same time, ghrelin levels were significantly lower compared with those with the wild type AA, i.e., Arg16/Arg16 genotype, and the difference was statistically significant (p< 0.05). No effect of this SNP was seen on the lipid levels (cholesterol, triglyceride, LDL-cholesterol, and HDL-cholesterol).

**Table 5:** The levels of the different demographic parameters, hormonal and lipid profiles in the different genotypes of rs1042713 (Arg16Gly) n = Number; SE = Standard error of the mean

Variables	AA (n=109) Mean±SE	AG (n=46) Mean±SE	GG (n=45) Mean±SE	P-value
BMI (kg/m^2^)	27.45±0.51	30.50±1.15	33.05±1.12	0.0001
Waist (cm)	82.43±1.24	89.0±2.69	93.42±2.55	0.0001
Hip (cm)	104.11±1.08	108.2±2.25	113.33±2.29	0.001
W/H ratio	0.789±.007	0.819±.014	0.822±.012	0.025
Cholesterol (mmol/L)	3.950±.073	4.096±.102	4.238±.132	0.102
Triglyceride (mmol/L)	0.971±.041	1.020±.062	1.014±.056	0.730
HDL cholesterol (mmol/L)	1.203±.032	1.171±.055	1.097±.039	0.199
LDL cholesterol (mmol/L)	2.034±.071	2.154±.101	2.283±.117	0.158
Leptin (ng/mL)	23.52±1.250	31.54±3.389	35.49±2.994	0.0001
Fast ghrelin (ng/mL)	0.439±.012	0.3843±.0212	0.3477±.0213	0.0001
Fasting insulin (pmol/L)	84.16±4.74	84.34±7.51	101.91±7.71	0.118
Fasting glucose (mmol/L)	4.854±.043	4.909±.077	5.067±.073	0.043

**Table 6:** The value of the demographic parameters, hormonal and lipid profiles in the different genotypes of rs1042714 (Gln27Gly) n = Number; SE = Standard Error of the mean

Variables	CC (n=121) Mean±SE	CG (n=28) Mean±SE	GG (n=51) Mean±SE	P-value
BMI (kg/m^2^)	29.04±.59	28.83±1.25	30.61±1.08	0.348
Waist (cm)	85.07±1.33	84.96±2.77	90.41±2.63	0.114
Hip (cm)	106.26±1.23	104.86±2.61	110.43±1.93	0.122
W/H ratio	0.798±.0068	0.808±.0164	0.814±.0136	0.503
Cholesterol (mmol/L)	4.036±.072	3.968±.125	4.120±.116	0.689
Triglyceride (mmol/L)	0.885±.031	1.077±.083	1.198±.065	0.000
HDL cholesterol (mmol/L)	1.225±.032	1.046±.0553	1.114±.040	0.012
LDL cholesterol (mmol/L)	2.118±.064	2.093±.137	2.130±.120	0.978
Leptin (ng/mL)	25.96±1.38	28.48±3.89	32.80±3.09	0.076
Fasting ghrelin (ng/mL)	0.421±.012	0.411±.029	0.368±.019	0.081
Fasting insulin (pmol/L)	84.55±4.82	91.64±8.67	94.96±6.58	0.435
Fasting glucose (mmol/L)	4.91±.0478	4.936±.080	4.912±.056	0.969

Table 6 shows there was no difference in the anthropometric data, lipid and hormonal profiles between the three genotypes (CC, CG, GG), except for the triglyceride, which was significantly higher and HDL-cholesterol which was significantly lower in the individuals with GG genotype.

Correlation studies were carried out between fasting glucose and the different parameters separately in the different genotypes of rs1042713 and rs1042714. Table 7 shows the correlation between glucose and the studied parameters in different genotypes of rs1042713. In the individuals with AA genotype (Arg/Arg), glucose correlates positively and signi ficantly with BMI, waist, WHR, cholesterol, triglyceride, LDL-cholesterol, insulin and leptin, and negatively with HDL-cholesterol and ghrelin. In the GG genotype (Gly/Gly), all correlations were lost, except the negative correlation with ghrelin and positive correlation with insulin. In the heterozygotes, the positive correlation with cholesterol and LDL-cholesterol became significant, and the negative correlation with ghrelin remained. All other relations were lost.

**Table 7:** Correlation between fasting glucose, and the anthropometric parameters and lipids in individuals with different genotypes (AA, AG, GG) of rs1042713 (Arg16Gly) (n) = Number; r = Correlation Coefficient; p = Significance

rs1042713 Genotype (n)	Correlation	Correlation between glucose and:										
		BMI	Waist	Hip	WH ratio	Cholesterol	Triglyceride	HDL-C	LDL-C	Leptin	Fasting ghrelin	Fasting Insulin
AA (109)	r	0.247	0.262	0.102	0.357	0.296	0.284	-0.202	0.312	0.192	-0.248	0.301
	p	0.010	0.006	0.289	0.000	0.002	0.003	0.035	0.001	0.046	0.009	0.001
AG (46)	r	0.130	0.189	0.176	0.097	0.383	0.058	0.189	0.508	0.260	-0.373	0.217
	p	0.390	0.209	0.242	0.520	0.009	0.702	0.208	0.000	0.081	0.011	0.147
GG (45)	r	0.127	0.129	0.124	0.060	-0.17	-0.04	-0.342	-0.032	0.098	-0.19	0.433
	p	0.405	0.397	0.416	0.697	0.274	0.781	0.021	0.836	0.523	0.216	0.003

Table 8 presents the correlation between glucose and the studied parameters in individuals carrying genotypes of rs1042714. For the CC genotype (Gln/Gln), a significant correlation was observed with the BMI, waist, W/H ratio, cholesterol, LDL-cholesterol, and fasting insulin. There was a negative correlation with ghrelin and no correlation with hip circumference, triglyceride, HDL-cholesterol, and leptin. In the GG genotype (Glu/Glu), glucose correlated positively with BMI, waist, W/H ratio, leptin, and insulin. In the heterozygotes (CG; Gln/Glu), there were no correlations between glucose and any of the studied parameters.

**Table 8:** Correlation between fasting glucose and the anthropometric parameters, lipids and hormones in individuals with different genotypes (CC, CG, GG) of rs1042714 (Gln27Glu; C>G) n = Number; r = Correlation Coefficient; p = Significance

rs1042714 Genotype (n)	Correlation	Correlation between glucose and:										
		BMI	Waist	Hip	WH ratio	Cholesterol	Triglyceride	HDL-C	LDL-C	Leptin	Fast ghrelin	Fasting insulin
CC(121)	r	0.213	0.234	0.126	0.295	0.280	0.114	-0.106	0.326	0.171	-0.313	0.337
	p	0.019	0.010	0.169	0.001	0.002	0.211	0.249	0.000	0.061	0.000	0.000
CG(28)	r	-0.116	-0.014	-0.0001	-0.006	0.062	0.168	-0.07	0.198	0.134	0.004	0.161
	p	0.557	0.945	0.996	0.975	0.756	0.394	0.729	0.312	0.498	0.984	0.414
GG(51)	r	0.353	0.341	0.242	0.358	0.136	0.209	-0.13	0.270	0.297	-0.35	0.325
	p	0.011	0.014	0.087	0.010	0.341	0.141	0.362	0.055	0.034	0.011	0.020

## Discussion

**Figure 1:**
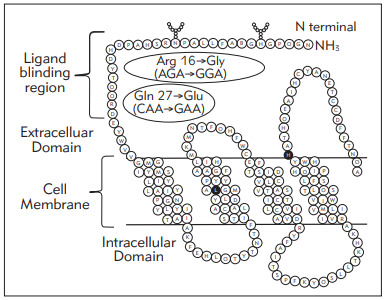
Part of the structure of ADRB2, showing the location of the two studied SNPs in the ligand-binding region

Several studies have been conducted on the PCOS patients in Saudi Arabia, as PCOS poses as a frequently identified abnormality in females of childbearing age [9] [10] [11]. Only a few of the earlier studies have explored the effect of *ADRB2* gene polymorphism in the etiology of PCOS and the influence of this polymorphism on the hormonal and biochemical parameters in PCOS patients. In this study, we investigated two *ADRB2* gene polymorphisms, and compared the genotype and allele frequencies in the PCOS patients and non-PCOS controls, both obese and normal weight. The ADRB2 was selected since several studies have shown that it plays an essential role in the expenditure of energy and the control of body weight. Polymorphisms in this gene are associated with the activation of the sympathetic nervous system and hence are involved in the pathogenesis of obesity, hypertension, insulin resistance, and other obesity-related disorders [12] [13] [14]. Our earlier studies have shown an association of ADRB2 with obesity and related disorders in the Saudi population [15] [16]. The ADRB2 is a G protein-coupled receptor, with an extracellular amino-terminal domain, seven transmembrane domains, and an intracellular carboxyl-terminal domain. Both SNPs investigated during this study are non-synonymous and result in changes in the amino acids flanking the receptor's ligand-binding site [17], as shown in Figure 1. Using site-directed mutagenesis and transfection experiments, studies have shown that the mutant Gly16 undergoes agonist-promoted down-regulation. On the other hand, the Glu27 variant shows a strong resistance towards both agonist-promoted desensitization and down-regulation, resulting from a modified susceptibility to receptor protein degradation.

We also compared anthropometric data, lipid, and hormonal parameters in the PCOS group and the control group. The results showed that the waist circumference and the WHR were significantly higher, while the hip circumference was lower in the PCOS group compared to the control group. It has been shown that the abdominal fat plays a vital role in the etiology of PCOS. Kaye et al. [18] and Hollmann et al. [19], reported that WHR of around 0.8 or higher was a factor playing a role in reproductive hormone disturbances, menstrual abnormalities and in increasing the prevalence of infertility.

A comparison of the lipid profile of total PCOS patients with the total control group showed several lipid abnormalities in the PCOS. As is well established, elevated waist and WHR, together with higher levels of cholesterol, triglycerides, LDL cholesterol, and lower HDL cholesterol levels, are major players in inducing cardiovascular abnormalities (CVD) [20]. Our results show that in both obese and non-obese PCOS, the predictive markers of CVD exist, and the PCOS are at a higher predisposition to cardiovascular abnormalities. Our findings are in line with those of Pirwany and coworkers [21], who also showed similar abnormalities in PCOS. We suggest that PCOS patients must be followed carefully to prevent the development of CVD and other related abnormalities. Many other studies have pointed to the significant association between lipid abnormalities and the development of metabolic syndrome [22], myocardial infarction, stroke, and high visceral fat [23].

Glucose levels were elevated in the total PCOS, whether normal weight or obese, compared to their healthy counterpart controls, and in each group, the difference was statistically significant. Many studies have reported glucose, glucose tolerance, and HbA1c abnormalities in PCOS group [24]. In this study, we excluded all PCOS patients suffering from diabetes mellitus; still, the difference in the glucose level in PCOS compared to the controls was statistically significant. The level of fasting plasma glucose has been considered as a suboptimal predictor of impaired glucose tolerance and diabetes mellitus [25]. It is also associated with adverse pregnancy outcomes [26] and is a marker of CVD risk [27]. Since these abnormalities occur frequently in patients suffering from PCOS, hence, the PCOS patients must be regularly monitored for glucose level, both fasting and postprandial [28].

This study also shows that insulin levels in the total, normal weight, and obese PCOS patients were higher, though the difference in the obese PCOS compared to obese control was not statistically significant. Extensive studies have monitored insulin levels in PCOS, and it is shown that the frequency of hyperinsulinemia and insulin resistance is high in PCOS [29]. Many abnormalities in PCOS are related to the abnormal insulin levels, and hyperinsulinemia is considered as an independent cardiometabolic risk factor [27].

In contrast to several reports, our results show that ghrelin and leptin levels do not differ when the total, normal weight and obese PCOS patients are compared to their control counterparts. Thus, suggesting that PCOS does not affect the level of these hormones. Both these hormones play an essential role in influencing energy balance. Leptin is an anorexigenic hormone and suppresses food intake and is believed to induce weight loss. A recent meta-analysis reported elevated leptin levels in PCOS [30], but the results of this study are in disagreement with the results of the meta-analysis.

Interestingly, when we compared the results in the normal weight PCOS with the obese PCOS, leptin levels were significantly higher. The same result was obtained when the normal weight controls were compared to the obese controls. We suggest that higher levels of leptin in the obese PCOS and obese control are a result of an increase in BMI and not due to PCOS as indicated by some workers [31]. Ghrelin, a fast-acting hormone, plays a role in meal initiation. In obese people, the ghrelin levels are significantly decreased compared to the normal weight. This was confirmed by our results, since in our obese PCOS and obese controls, the levels were lower than in the normal weight PCOS and normal-weight controls, respectively. We suggest that this is an effect of BMI on ghrelin and not the effect of PCOS, as suggested in a meta-analysis on ghrelin levels in PCOS, which showed a decrease in ghrelin level [32]. It is possible that other genetic/environmental/epigenetic factors also contribute to controlling leptin and ghrelin levels.

The results of the two studied SNPs in the *ADRB2* gene showed that rs1042713 is not an etiological factor in the development of PCOS (p>0.1). Even when the obese and normal-weight groups were separated and compared with their control counterparts, no differences were identified. These results in Saudis are in agreement with results from a Japanese population that did not report any association between this polymorphism and PCOS [6]. However, our results show a significant association of genotypes of this SNP with the anthropometric, lipid, and hormonal parameters. The GG genotype of this SNP had a substantial effect on the BMI, hip, waist circumference, W/H ratio, leptin, and ghrelin levels, where these parameters were elevated in the GG group, except ghrelin, which was significantly reduced. The hyperleptinemia was significant in the GG genotype. There was a small effect on the lipid levels, and the GG genotypes had higher lipid levels, but there were no significant differences. Our earlier study on the Saudi population showed that rs1042713 is linked to obesity, hyperlipidemia, and hyperleptinemia [15]. In studies reported from Korean and Colombian populations, this SNP was not related to either bodyweight or lipid abnormalities [33] [34]. However, several other studies have shown that this SNP is linked to lipid and body weight abnormalities [35] [36] [37]. This SNP has also been linked to the structure and function of the cardiovascular system, neuronal, hormonal activation, and exercise tolerance [38]. These findings highlight the need to investigate the role of rs1042713 and rs1042714 in each population, since these genetic loci may influence the development of several associated complications.

The SNP rs1042714 shows association with PCOS, where the prevalence of CC genotype and C allele was higher compared to the controls, and the difference almost reached significance (c^2^=3.692; p=0.054). When the allele frequency in the normalweight controls was compared with the normal weight PCOS, a statistically significant difference (c^2^ = 4.238; p=0.039) was observed, though there was no difference in the obese group. Earlier studies have linked the G allele to elevated triglycerides and leptin in the normal population [16] [39]. In this study, the most apparent abnormality in the GG genotype was high triglyceride levels and decreased HDL-cholesterol level.

Glucose was correlated to the different studied parameters, and it was interesting to note that in different genotypes of the two studied SNPs, the correlation pattern between glucose and other parameters differed. The correlation of the anthropometric, lipid, and hormonal parameters with an increase in glucose level, depended on the genotype of rs1042713. The most significant correlations were in the individuals carrying the AA genotype, where the BMI, waist, WHR, cholesterol, triglyceride, LDL cholesterol, and fasting insulin, all positively correlating with glucose, while HDL-C and fasting ghrelin correlated negatively. On the other hand, in individuals with GG and AG genotypes majority of these correlations were lost. This finding suggests that the manner in which a nutrient influences the development of a complication is subject to the effect of the genotype of the different SNPs, he/she is carrying. We further suggest that the contributions from other genes or polymorphisms may modify the clinical presentation further. This suggestion requires confirmation.

This is the first study on rs1042713 and rs1042714, in the *ADRB* gene in Saudi PCOS females, and the major limitation of this study is the small number of PCOS patients and controls. In the future, more extensive studies are recommended to strengthen the conclusions of the present study.

In conclusion, it is worth pointing out that populations differ in the frequencies of different polymorphic variants and hence in the clinical presentation of a particular disease, including multifactorial disorders. These differences result from the metabolic variations that exist in patients carrying different genotypes of a mutation/polymorphism. Thus, it is essential point to conduct such studies in each population and to come up with care and treatment strategies, which are population-specific and which can later be individualized.

### Consent for publication

The authors have no objection to the publication of this study.

### Competing interests

The authors declare that they have no competing interests.

### Funding

The study was supported by the ‘Deanship of Scientific Research, King Saud Universities, Riyadh, Saudi Arabia, for funding this work through research group No. RG-1440-077.

### Acknowledgements

The authors extend their appreciation to the Deanship of Scientific Research, King Saud Universities, Riyadh for funding this work through research group No. RG-1440-077. We thank all the subjects for their cooperation and participation in the study. We would also like to thank all the participants (researchers, technicians and nurses) for their notable contribution.

## Conflict of interest statement

The authors declare that they have no conflicts of interest in this work.

## List of abbreviations

PCOS, Polycystic ovarian syndrome; CVD, Cardiovascular disease; ADRB2, Beta-2-adrenergic receptor gene; SNP, Single nucleotide polymorphism; HDL-C, High-density lipoprotein (HDL)-cholesterol; LDL-C, Low-density lipoprotein (LDL)-cholesterol; BMI (W/H2), Body mass index (Weight/Height2); IRB, Institutional Review Board; WHR, Waist-hip ratiol; EDTA, Ethylenediamine tetraacetate; ELISA, Enzyme-linked immunosorbent assay; ECLlA, electrochemiluminescence immunoassay; SEM, Standard error of the mean; CI, Confidence Interval; HbA1c, Hemoglobin A1c
